# GC–MS analysis of volatile organic compounds from Bambara groundnut rhizobacteria and their antibacterial properties

**DOI:** 10.1007/s11274-019-2660-7

**Published:** 2019-05-27

**Authors:** Caroline F. Ajilogba, Olubukola O. Babalola

**Affiliations:** Food Security and Safety Niche Area, Faculty of Natural and Agricultural Science, North-West University, Mmabatho, Mafikeng, 2735 South Africa

**Keywords:** Antibacterial, Bambara groundnut, GC–MS, Secondary metabolites, Volatile organic compounds

## Abstract

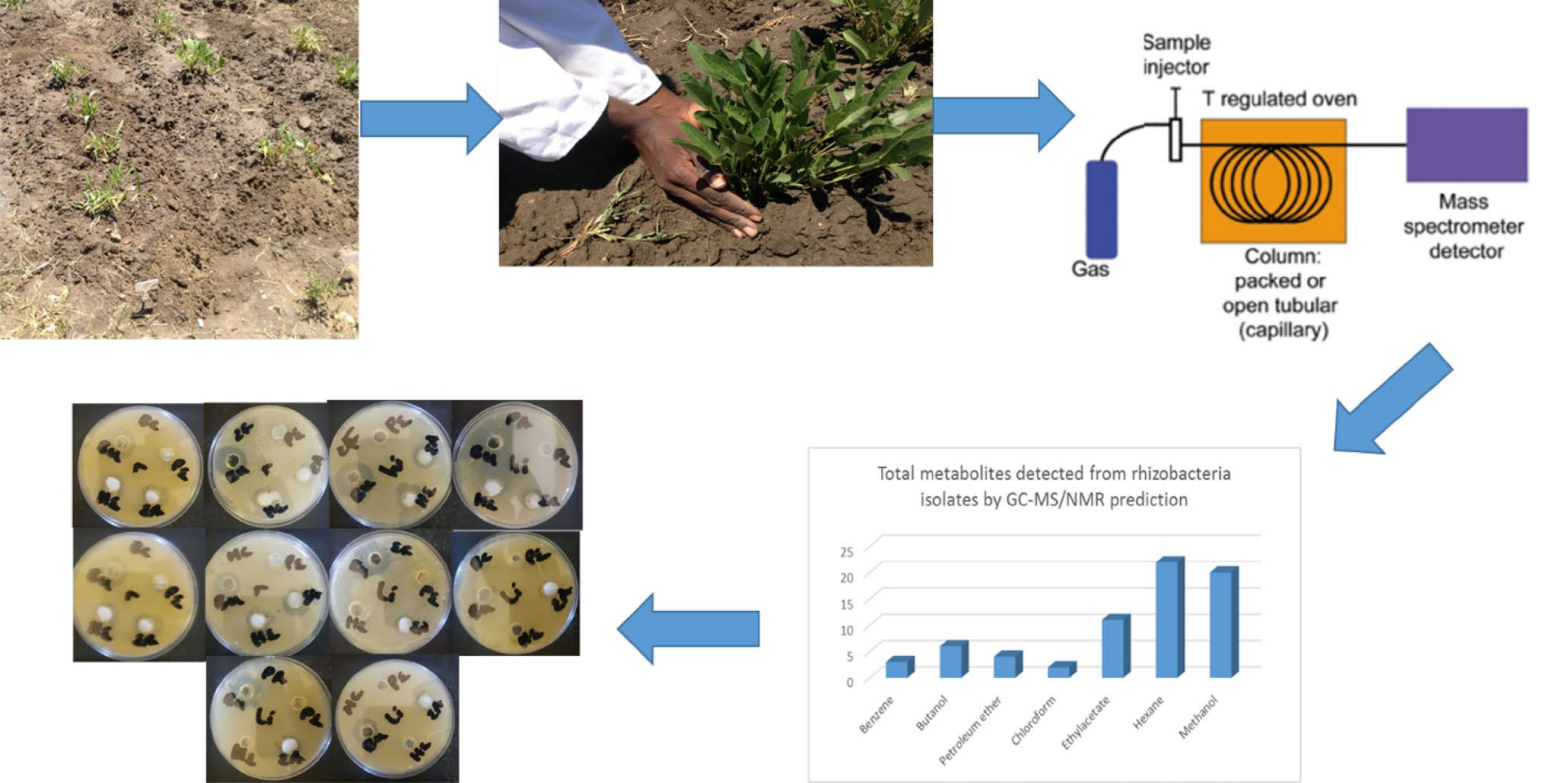

## Introduction

Many rhizobacterial species are involved in plant growth promotion (PGP) and biocontrol activities. Diverse mechanisms are applied directly and or indirectly such as indole acetic acid production and phosphate solubilisation which have been used to achieve this PGP potential (Ajilogba and Babalola 2013). Others are able to suppress or antagonise the growth of competing or pathogenic microbes that can cause disease in or/and around the plant by the production of hydrogen cyanide (HCN), siderophores, antibiotics and other antibacterial compounds such as 2,4-diacetylphloroglucinol and phenazine derivatives (Szentes et al. 2013).

Rhizobacteria are recognized as potential sources of bioactive substances and they are therefore involved in the production of secondary metabolites in the rhizosphere (Kanchiswamy et al. 2015). Rhizobacteria from leguminous crops are important producers of volatile organic compounds (VOCs). As natural nitrogen-fixers, they help trap atmospheric nitrogen and make it available to plants for plant growth and development. Hydrogen cyanide is a volatile compound and an antibiotic effective against other pathogenic organisms and roots of weeds (Ali et al. 2015). It is a common metabolite found in 50% of *Bacillus* and over 88% of *Pseudomonas* in the rhizospheric soil and plant root nodules (Ahmad et al. 2008).

Bambara groundnut is important in Agriculture and an extremely drought-tolerant crop (Masindeni 2006). It can perform well and have good crop yield on marginal soils and soils that have undergone water stress compared to other legumes (Brink et al. 2006). It grows well even in poor and infertile soils (Opoku 2010). Also, it can grow on soils of both high and low nitrogen content and high and low temperature conditions (Baudoin and Mergeai 2001). Because it tolerates poor soil, farmers with poor resource especially in funding of fertilizer to increase yield are encouraged to farm more with Bambara groundnut. Report shows that the ability of Bambara groundnut to tolerate poor soil is advantageous against being planted in nitrogen-rich soil. This is because nitrogen-rich soils increase vegetative growth of Bambara groundnut as against crop pod and seed productivity (Baudoin and Mergeai 2001). As a legume, root nodules bacteria form symbiotic association with Bambara groundnut roots. This helps to increase the nitrogen content of the soil in the sense that the bacteria assimilate atmospheric nitrogen, trap it and make it available to the plant in the soil. This process in turn helps to increase soil fertility and crop yield (Masindeni 2006). It has a high tolerance to disease and pest (Ajayi and Lale 2001).

Rhizobia species (*Rhizobium*, *Bradyrhizobium*, *Azorhizobium*, *Allorhizobium*, *Sinorhizobium* and *Mesorhizobium*) have been known to suppress growth of plant pathogens and also form nodules in symbiotic relationship with legumes (Dakora 2003). The symbiotic relationship also results in the production of nitrogen rich soil. Bambara groundnut was found to form nodules and fix nitrogen in partnership with *Bradyrhizobium* strain (Laurette et al. 2015). Nodule formation is important in Bambara-microbe interaction; this process starts with production of compounds such as betaines, flavonoids and aldonic acid in the root exudates of the plants. These compounds then have to signal to the rhizobia in a compatible relationship with the compounds. This in turn enhances the production of the nod gene that induces nodulation by interacting with the nodD protein of the cell wall of the rhizobia (Phillips 2000). The rhizobia react to this inducement by producing and releasing the lipo-chito-oligosaccharide Nod factors, which bring about morphological changes in the root hair of the legume. This leads to the formation of an infection thread and development of nodules that finally enhances fixation of nitrogen (Dakora 2003). Some of the molecules found in root exudates/flavonoids of Bambara groundnut include genistein, coumestrol and daidzein (Dakora 2000). The genera involved in secondary metabolite production in the rhizosphere include *Pseudomonas*, *Bacillus*, *Klebsiella*, *Azotobacter*, *Azospirillum*, *Azomonas, Rhizobium*, *Bradyrhizobium* and *Mesorhizobium* (Ahemad and Kibret 2014). Secondary metabolites secreted by rhizobacteria are involved in different biological activities such as antimicrobial, antiprotozoal, antihelmintic, antitumor, anticancer, antifungal and immunosuppressant (Graça et al. 2013; Sadrati et al. 2013).

*Bacillus* spp. have been known to secrete secondary metabolites such as antifungal, antibacterial and siderophores. Antibiotics produced by *Bacillus* spp. include a wide variety of bacteriocin and have been found to be important as antibiotic precursors, biocontrol agents of plant pathogens and as biopreservatives of beverages and food systems (Sansinenea and Ortiz 2011). An important example of bacteriocin includes megacin secreted by *B. megaterium*. Khalil et al. (2009) observed that bacteriocin was produced and secreted by *B. megaterium* in both solid and liquid medium and it was able to inhibit the growth of pathogenic bacteria thereby serving as a biopreservative.

Hydrogen cyanide and 2,4-DAPG were observed to function as nematicides and contributed to the toxicity of the bacteria *P. fluorescens* CHA0 against nematode *Caenorhabditis elegans* by repelling the nematode (Neidig et al. 2011)*.* Bacilysin, a simple peptide antibiotic was observed to possess both antifungal and antibacterial properties. It is a biocontrol agent against *Erwinia amylovora*. It was isolated from several strains of *B. pumilus*, *B. subtilis* and *B. amyloliquefaciens* (Arguelles-Arias et al. 2009; Steinborn et al. 2005). Other secondary metabolites have been documented to be secreted by rhizobacteria from different crops for biocontrol and plant growth activities.

Volatile organic compounds (VOCs) are secondary metabolites produced by soil and plant-associated microorganisms, but largely unexplored to date. They are typically small, odorous compounds ( < C15) with low molecular mass ( < 300 Da), high vapor pressure, low boiling point, and a lipophilic moiety. These properties facilitate evaporation and diffusion aboveground and belowground through gas- and water- filled pores in soil and rhizosphere environments (Schulz-Bohm et al. 2017). Also, these bioactive substances and secondary metabolites emitted from the rhizobacteria of Bambara groundnut have not been studied. The main aim of the study was to identify and extract bioactive compounds with the use of diverse solvent and then assay for their antibacterial activities.

## Materials and methods

### Soil sampling and collection

Soil samples were collected from field trials during the planting period between October 2014 and March 2016 from the North-West University Agricultural Farm, Mafikeng Campus (Lat. 25°78ʹ91ʺ Long. 25°61ʹ84ʺ) Mafikeng, South Africa. Four soil samples were collected randomly from the uprooted Bambara groundnut root rhizosphere at four weeks interval from bulk soil before planting for 16 weeks corresponding to different growth stages of the plant. Twenty four (24) soil samples were collected in all to a depth of 15 cm per sample. Samples were collected in triplicates stored at 4 °C until ready for use.

### Preparation of soil samples for bacterial isolation

Soil samples from the rhizosphere of Bambara groundnut were collected. Samples were prepared according to Abdulkadir and Waliyu (2012) and placed into sterile 50-ml Falcon tubes (Becton Dickson Paramus, N.J) and kept on ice or at 4 °C until it was needed (within 3 days).

### Culturing and isolation of bacteria from samples

Isolation and enumeration of bacteria present in the soil sample were performed by serial dilution plate technique using tryptone soy agar (TSA). A tenfold dilution series was prepared from 10 g of the rhizospheric soil of Bambara groundnut in sterile distilled water and 0.5 ml from the selected dilution was spread plated on the already set tryptic soy agar (TSA). Two (2) loopfuls of each of the bacteria from 3-day old cultures on TSA were each transferred separately to 50 ml tryptic soy broth (TSB) medium and incubated overnight at 28 ± 2 °C. A loopful from each TSB bacterial inoculum was streaked on prepared TSA to have pure isolates from each broth culture.

### Molecular identification of selected isolates

Bacterial universal primers (F1R2) amplified 1.5 kb fragment from the genomic DNA of the isolates. Computational analysis was used as a means of identifying the isolates. Analysis of the partial sequences of the 16S rDNA gene of the selected isolates was used as a means of identifying them at the genus level. The BLAST tool was used to compare the partial nucleotide sequences of the 16S rDNA gene of the isolates with the nucleotide database of NCBI web server.

The 16S rDNA gene sequence of the selected isolates was obtained by BLASTn search. Table1 results show that query sequences were best pairwise aligned with 16S rDNA gene sequence of other firmicutes, proteobacteria and actinobacteria with sequence similarity and identity ranged between 96 and 99%, with E-value of 0.

**Table 1:** Results of 16S rDNA gene sequence similarities of bacterial isolates and GenBank accession numbers using BLASTn algorithm

Isolate code	Sequence length (bp)	Closest related strain in database	Accession number	Similarity (%)	E-value
BAMbi	1403	*B. safensis*	KX809651	99	0
BAMhi	1443	*B. amyloliquefaciens*	KX809652	99	0
BAMli	1388	*B. thuringiensis*	KX809653	99	0
BAMr	1058	*Bacillus* sp.	KX588095	98	0
BAMpii	1414	*M. hydrocarbonoxydans*	KX809654	99	0
BAMrii	1416	*M. testaceum*	KX809655	99	0
BAMxii	1389	*Acinetobacter parvus*	KX809656	98	0

### Antagonism assay against phytopathogenic fungi and bacteria

Potato dextrose agar (PDA) medium was used as the medium to assay for the antifungal activities of 8 isolates against *Fusarium graminearum*. This was carried out by inoculating the pathogenic fungi at the centre of the medium and then streaking the isolates on the medium 3 cm away from the fungi. The clear zones between isolates and fungi after incubation for 4 to 7 days at room temperature indicated antagonist interaction between them.

Antagonistic activity of isolates against Gram-negative and Gram-positive bacteria was screened by using perpendicular streak method (Parthasarathi et al. 2010). In perpendicular streak method, Luria Bertani agar (Merck) was used and each plate was streaked with test bacteria isolates at the centre/diameter of the plate and incubated at 30 °C for 48 h to allow optimum growth. Later, 24 h fresh sub-cultured isolated bacteria were prepared and streaked perpendicular to the test isolates and incubated at 37 °C for 24 h. The experiment was carried out in triplicate.

Perpendicular streak method was used to rapidly screen microbes for antagonism (Lertcanawanichakul and Sawangnop 2011).

### Cultivation of the isolates for extraction of bioactive compounds

Out of the 8 isolates, 3 *Bacillus* spp. (*B. amyloliquefaciens*, *B. thuringiensis* and *Bacillus* sp.) were selected for cultivation based on reviewed literatures on their antagonistic activities. Suitable dilutions (10^–2^, 10^–4^, and 10^–6^) of the isolates were maintained on Luria Bertani (LB) agar medium (Merck) were used to isolate rhizobacteria. All plates (in three replicates) were incubated at 28 °C for 48 h. Each actively growing pure culture of the isolates was used to inoculate 100 ml of Luria Bertani broth (Merck) in a 250-ml Erlenmeyer flask.

### Extraction and partial purification of the crude extracts from the rhizobacteria isolates

After 48 h incubation at 30 °C, the LB broth (10%) was used to seed culture in three 500-ml Erlenmeyer flasks each containing 100 ml of fermentation medium (glucose, oatmeal, yeast extract, NaCl, CaCO3 at pH 7.0). The cultures were incubated at 25 °C for 10 days under constant agitation of 220 rpm. The fermentation medium was centrifuged for 20 min at 8000 × g to remove the bacterial cells. The supernatant was shared into 7 equal volumes of 10 ml each depending on the number of solvents used, and then each was extracted with 60 ml of organic solvent. A range of extraction solvents (7) was screened for effectiveness, including petroleum ether, *n*-hexane, chloroform, ethyl acetate, benzene, ethanol and *n*-butanol. From the extracts, 1 µl was used for the biological assay.

### Biological assay

The different test organisms were spread on plates and holes were bored. Each hole was filled with 1µl of each solvent extracts from the isolates Inhibition zones were measured after 24 h incubation at 37 °C. Agar-well diffusion method was used for the microbial extracts as it is widely used to evaluate the antimicrobial activity of plants or microbial extracts (Magaldi et al. 2004; Valgas et al. 2007).

### Gas chromatography-mass spectrometry (GC–MS) analysis

The partially purified active fractions were analyzed by GC–MS. The analyses of the compounds in the active fractions were run on a GC–MS system (Agilent GC: 6890, with a 7683B Autosampler). The fused-silica Rxi-5Sil MS capillary column (30 m 0.25 mm ID, film thickness of 0.25 mm) was directly coupled to an Agilent variant. Oven temperature was programmed (35 °C for 5 min, then 35–300 °C at 10 °C/min) and subsequently, held isothermal for 20 min. The injector port; was 250 °C, the transfer line: 290 °C, splitless. Volume injected: 0.2 ml and the column flow rate was 1 ml/min of 1 mg/ml solution (diluted in chloroform). The peaks of components in gas chromatography were subjected to mass-spectral analysis.

The MS was a LECO Pegasus 4D recording with a EI-source at − 70 eV; the solvent delay was 9 min. Scan time 1.5 s; acquisition rate 10 spectra/second; mass range 50–1000 amu; detector voltage 1800 V, and Ion source temperature: 250 °C. Data were recorded in TIC mode. The software adopted to handle the mass spectra and chromatograms was an Agilent chemstation software. The constituents were identified after comparing with available data in the GC–MS library in the literatures.

### Mass spectrometer

The GC–MS mass spectrum data were analyzed using Mnova 11.0.1 and the database of National Institute Standard and Technology (NIST) was used to interpret analyzed data. Comparison of the mass spectrum of the unidentified components released by the bacterial isolates was carried out against the mass spectrum of already known components available in the NIST library. The name, molecular weight and peak area percentage of unknown compounds were evaluated by the software as observed from the chromatogram. The name and structure of the components of the test materials were confirmed.

## Results

### Cultivation of rhizobacteria isolates

Three rhizobacterial isolates used in the field were BAMhi, BAMli and BAMr. These have been identified as *Bacillus* strains and they have been deposited in the GenBank (Table 1). BAMhi, BAMli and BAMr were identified as *Bacillus amyloliquefaciens*, *Bacillus thuringiensis* and *Bacillus* sp. respectively.

### Antimicrobial bacterial assay of identified Bacillus isolates against phytopathogenic fungi and bacteria

**Fig. 1:**
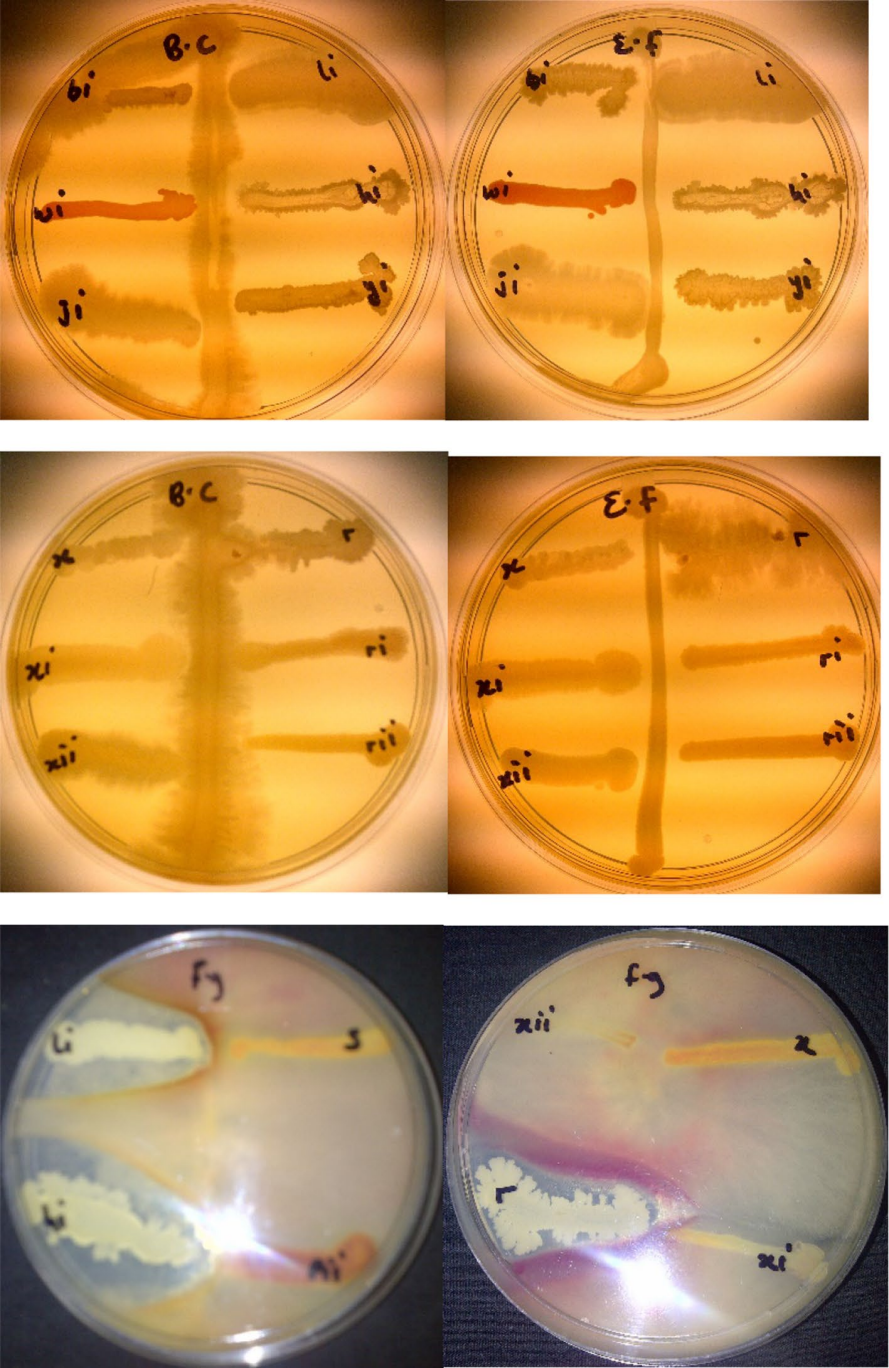
Antimicrobial assay of *Bacillus amyloliquefaciens* (hi), *Bacillus thuringiensis* (li) and *Bacillus* sp (r) against *Bacillus cereus* (BC), *E. feacalis* (EF) and *Fusarium graminearium* (fg)

The test organisms used in this assay are *B. cereus*, *E. feacalis* and *Fusarium graminearium* which all cause infections in man, animals and plants with high mortality rates. Antagonism against phytopathogenic fungi and bacteria is observed by isolated rhizobacteria *Bacillus amyloliquefaciens*, *Bacillus thuringiensis* and *Bacillus* sp. (Fig. 1).

### GC–MS analysis carried out on the rhizobacteria isolates

**Fig. 2:**
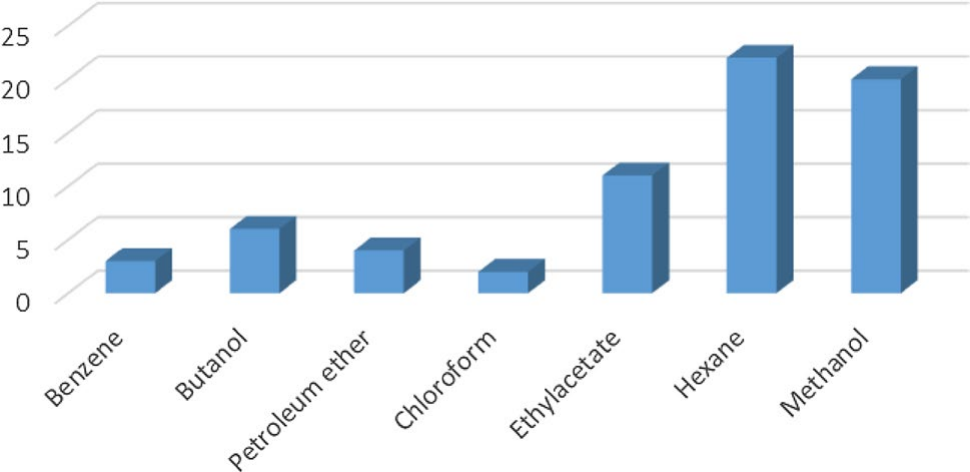
Total number of metabolites detected by GC–MS in *B. thuringiensis*, *B. amyloliquefaciens* and *Bacillus* sp. using seven different extraction solvents

The GC–MS results revealed that from the three identified *Bacillus* strains, *Bacillus amyloliquefaciens*, *Bacillus thuringiensis* and *Bacillus* sp., 31 VOCs were identified from isolate *B. thuringiensis* which was highest number of metabolites from the seven different extraction solvents used. *B. amyloliquefaciens* had the least with 15 VOCs identified while isolate *Bacillus* sp., had 22 VOCs identified (Table 3). With *B. Amyloliquefaciens*, the solvent with the highest metabolites is hexane with 5 VOCs while benzene and chloroform were the least with 1 VOC each. With *B. thuringiensis*, the solvent with the highest VOC was methanol having 15 VOCs while chloroform was the least with only 1. In *Bacillus* sp., the solvent with the highest VOCs was also hexane with 13 while benzene and chloroform had no hits. This shows that hexane detected the highest number of VOCs (22) from the 3 isolates while chloroform detected the least (2) while a total of 68 VOCs were detected from the isolates (Fig. 2 and Tables 3, 4, 5) in which case none of them has been identified before from the bacterial isolates of Bambara groundnut rhizosphere but some have been identified from rhizobacteria of other plants.

Some VOCs were commonly produced by the three isolates and they include tropone, *p*-xylene, ethylbenzene, carbazic acid and, isocarbaxazide (5). Others common to *B. thuringiensis* and *B. amyloliquefaciens* are formic acid butyl ester, paraldehyde and dimethylfulvene (3). Those common to *B. amyloliquefaciens* and *Bacillus* sp. are 2, 2-dimethylhexane-3-one and formic acid 2-methylpropyl ester (2). While those common to *B. thuringiensis* and *Bacillus* sp. are fumaronitrile, phenylethylalcohol, benzyl 2-chloroethyl sulfone, *N*-hydroxylmethylmethyl-2-phenylacetamide, tropeolin, benzene butyl, *n*-propylbenzene, phthalan, carbobenzoxyhydrazide and 1, 2-dimethylbenzene (10). Only 15 were not common across the 3 isolates and from *B. amyloliquefaciens*, they are, s-(+)-1, 2 propanediol and 2(3H)-furanone dihydro-5-methyl (2). From *B. thuringiensis*, they are, 2, 2-dimethylhexanone, 2-oxopropanoic acid, 2, 4-dimethylhexane, dihexylether, tridecylamine, 2, 4-dimethylhexane, cetane and hexane, 1, 1′-oxybis (8). Also from *Bacillus* sp., they are acetic acid butyl ester, isooctanol, benzene, tridecane and 1, 3-dimethylbenzene (5). (Tables 2, 3, 4).

**Table 2:** Bioactive compounds identified from GC–MS chromatogram of *B. amyloliquefaciens* (methanol, benzene, hexane, ethyl acetate, butanol, chloroform extract)

Extraction solvents	S/N	Retention time	Compound	Peak area %
Chloroform	1	5.4	Trichloromethane	27.37
Ethyl acetate	1	3.46	Paraldehyde	83.79
	2	5.05	Ethylbenzene	52.37
	3	5.35	Dimethylfuvene	89.11
	4	6.14	*p*-Xylene	47.92
Butanol	1	5.64	Formic acid butyl ester	19.98
	2	7.72	2,2-Dimethylhexane-3-one	41.08
Benzene	1	5.45	Tropone	24.06
Hexane	1	3.06	Phenylethylalcoholphenylethylalcohol, benzyl 2-chloroethyl sulfone, *N*-hydroxylmethylmethyl-2-phenylacetamide, tropeolin, benzene, butyl-, *n*-propylbenezene, phthalan and carbobenzoxyhydrazide	86.72
	2	3.47	2(3H)-Furanone dihydro-5-methyl	9.65
	3	3.76	Formic acid 2-methylpropyl ester	6.25
	4	5.05	Ethylbenzene, carbazic acid, isocarboxazid	37.72
Methanol	1	3.28	Paraldehyde	56.22
	2	3.47	s-(+)-1,2 propanediol	71.53

**Table 3:** Bioactive compounds identified from GC–MS chromatogram of *B. thuringiensis* (methanol, benzene, hexane, petroleum ether, ethyl acetate, butanol, chloroform extract)

Extraction solvents	Retention time	Compound	Peak area %
Chloroform	4.25	Bromodichloromethane	43.38
Ethyl acetate	3.46	Paraldehyde	83.57
	4.45	2-Oxopropanoic acid	41.55
	5.05	Ethylbenzene	52.37
	5.35	Dimethylfulvene	89.11
	6.14	*p*-Xylene	47.92
Butanol	5.36	Formic acid butyl ester	15.88
	7.7	2, 2-Dimethylhexanone	43.7
Benzene	4.37	Fumaronitrile	35.44
	5.34	Tropone	25.23
Hexane	5.03	Ethyl benzene	26.98
	3.57	Tridecylamine	12.76
	3.47	2,4-Dimethylhexane and dihexylether	55.54
Petroleum ether	3.47	2,4-Dimethylhexane	56.58
	5.03	Ethylbenzene	25.79
Methanol	3.28	Paraldehyde	56.22
	3.47	s-(+ )-1,2 propanediol	71.53

**Table 4:** Bioactive compounds identified from GC–MS chromatogram of *Bacillus* sp. (methanol, benzene, hexane, petroleum ether, ethyl acetate, butanol, chloroform extract)

Extraction solvents	Retention time	Compound	Peak area %
Ethyl acetate	5.35	*p*-Xylene	64.33
	6.15	1,2-Dimethylbenzene	36.89
Butanol	3.84	Acetic acid, butyl ester	60.41
	7.69	2,2-Dimethyl-3-hexanone	47.28
Hexane	3.06	Phenylethylalcoholphenylethylalcohol, benzyl 2-chloroethyl sulfone, *N*-hydroxylmethylmethyl-2-phenylacetamide, tropeolin, benzene, butyl-,* n*-propylbenezene, phthalan and carbobenzoxyhydrazide	86.72
	5.05	Ethylbenzene, isocarboxazid and carbazic acid	32.23
	3.47	Isooctanol	7.46
	3.76	Formic acid, 2-methylpropyl ester	8.78
Petroleum ether	3.48	Tridecane	61.72
	5.34	1,3-Dimethylbenzene	52.57
Methanol	3.81	Fumaronitrile, tropone and benzene	34.22

### Analysis of GC–MS chromatograms for *B. amyloliquefaciens*

#### Benzene and Butanol extract

**Fig. 3:**
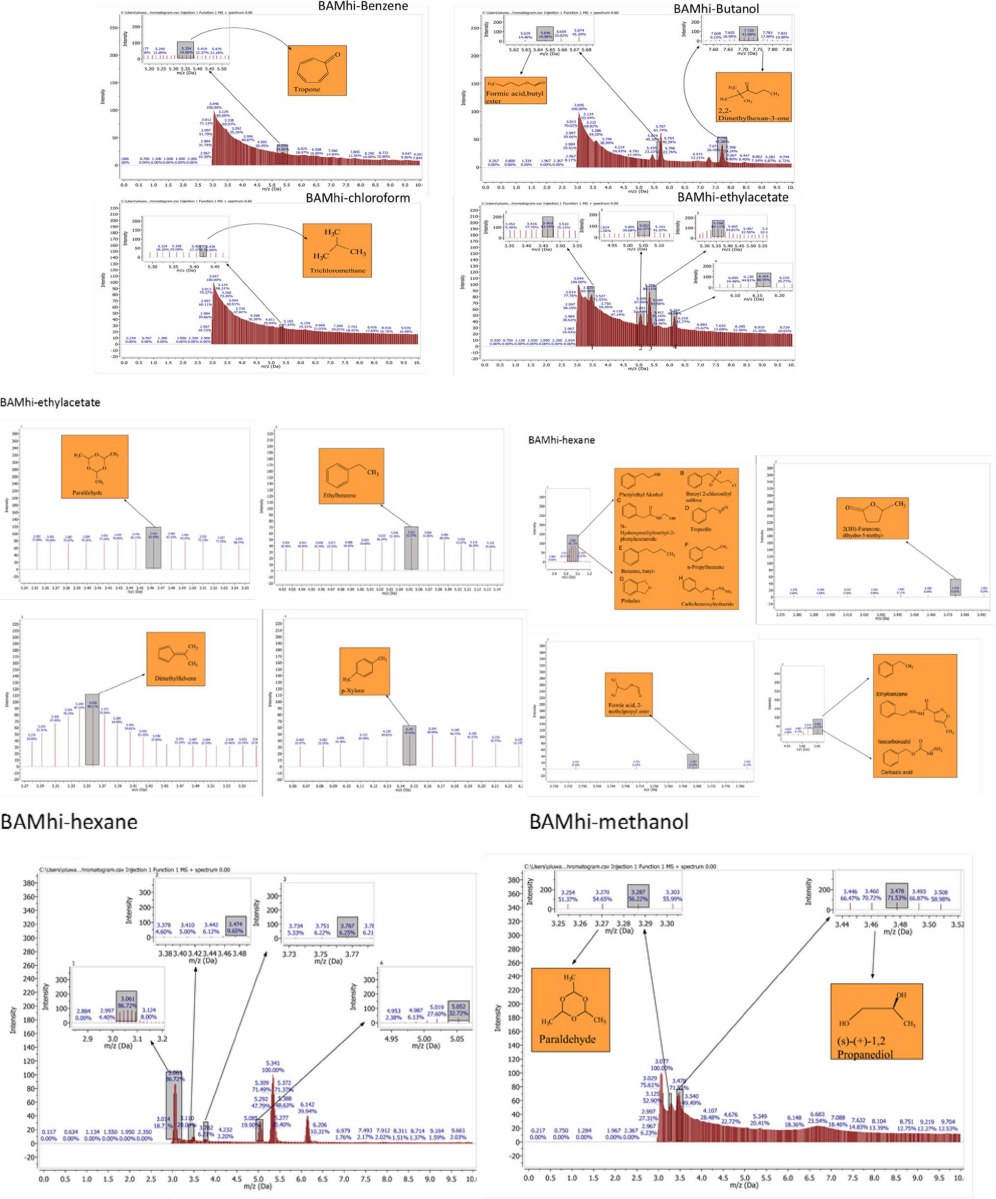
GC–MS chromatogram of *Bacillus amyloliquefaciens* (BAMhi) comparing extracts using benzene, butanol, chloroform, ethylacetate, methanol and hexane

**Fig. 4:**
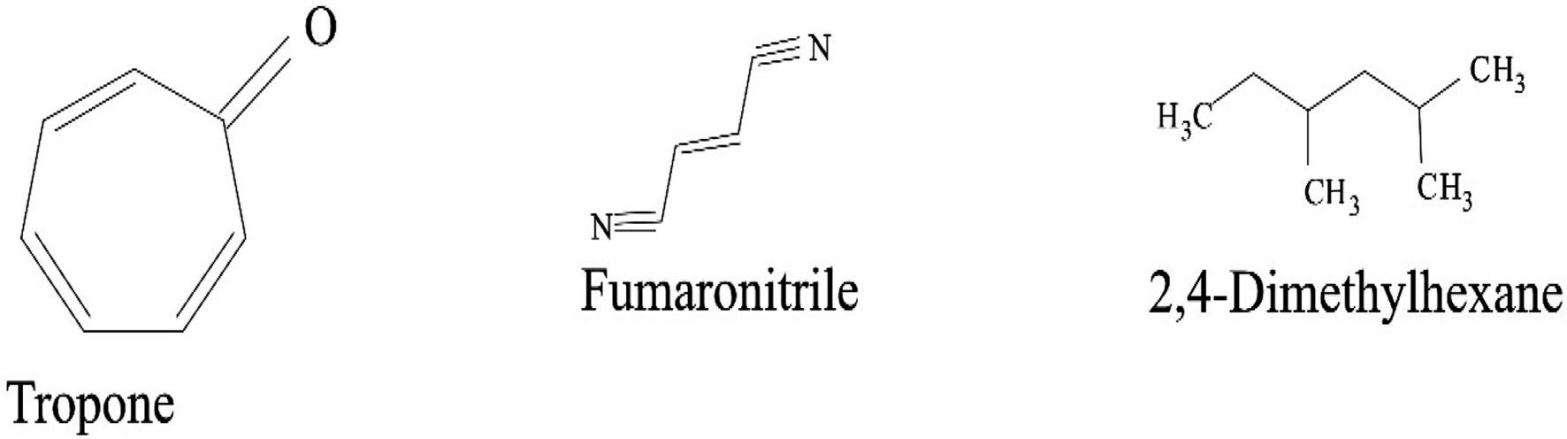
Structures of compounds from benzene fraction for all isolates

**Fig. 5:**
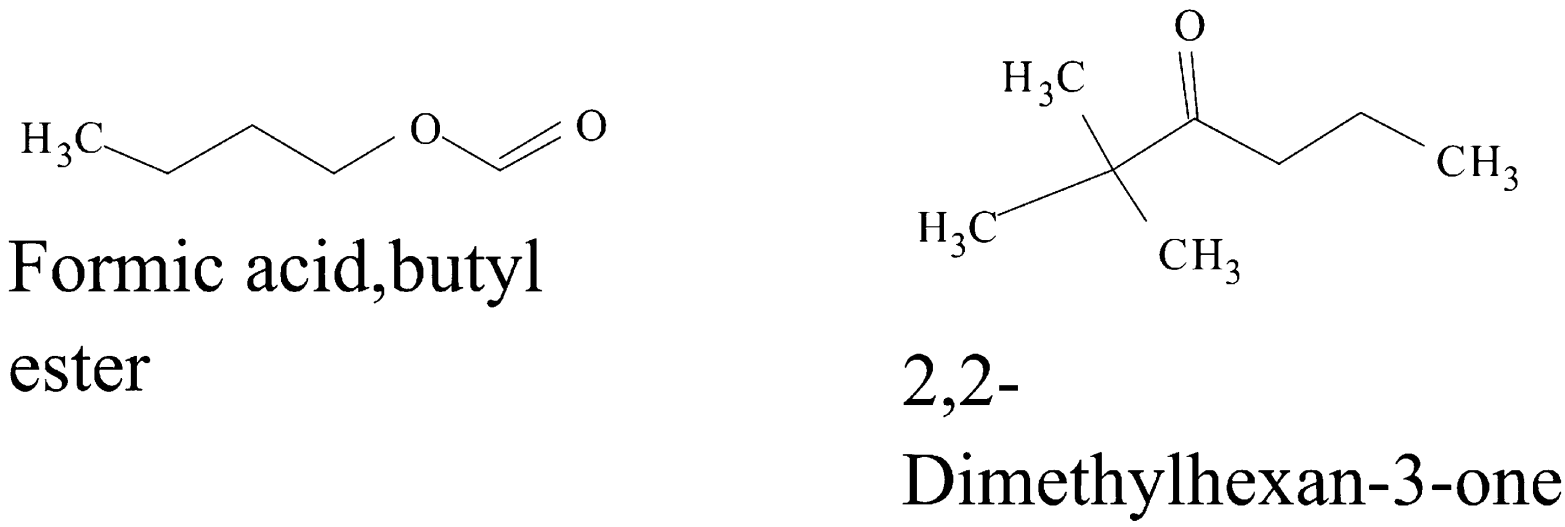
Structures of compounds from butanol fraction for all isolates

The GC–MS chromatograms of *B. amyloliquefaciens* benzene extract reveals a prominent peak which correspond to tropone on comparison with the mass spectra of the NIST library. Its intensity or peak area was 24.06% and 5.45 retention time (RT). The characterized peak and RT of the VOCs from butanol crude extract that is formic acid butyl ester and 2, 2-dimethylhexane-3-one were 19.98%/5.64 and 41.08%/7.72 respectively (Table 2, Figs. 2, 3, 4, 5).

#### Chloroform and ethyl acetate extract

**Fig. 6:**
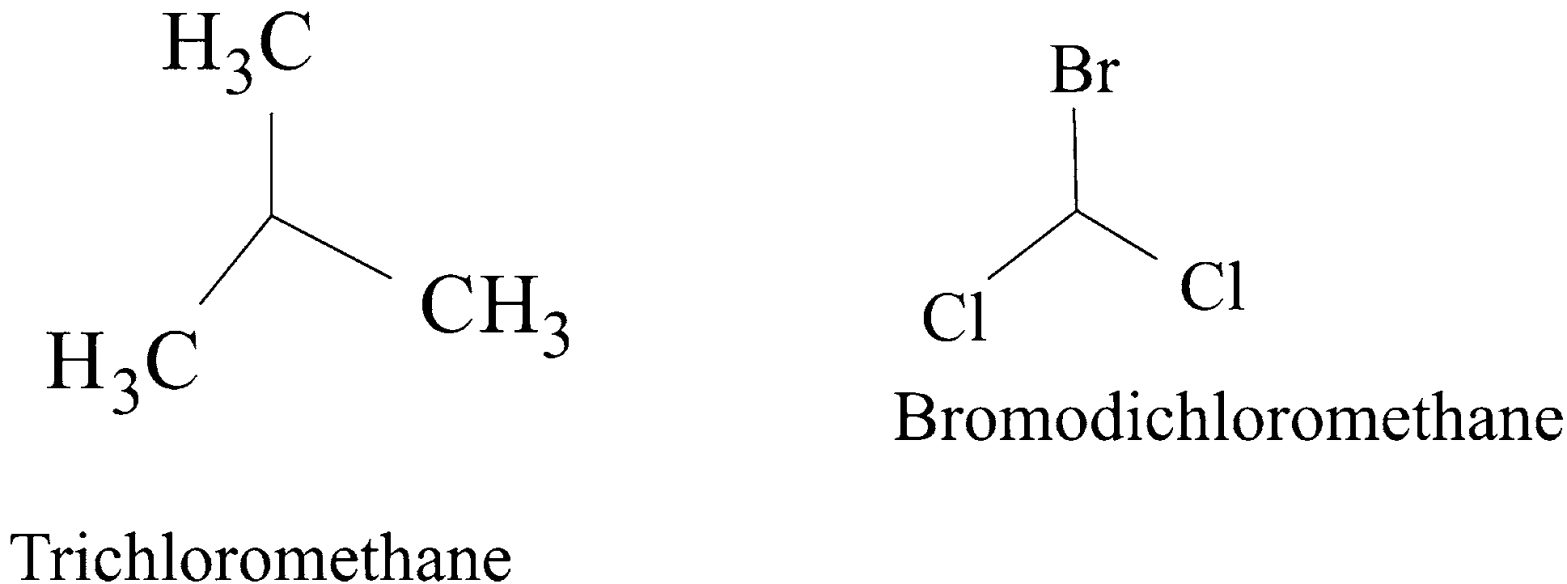
Structures of compounds from chloroform fraction for all isolates

**Fig. 7:**
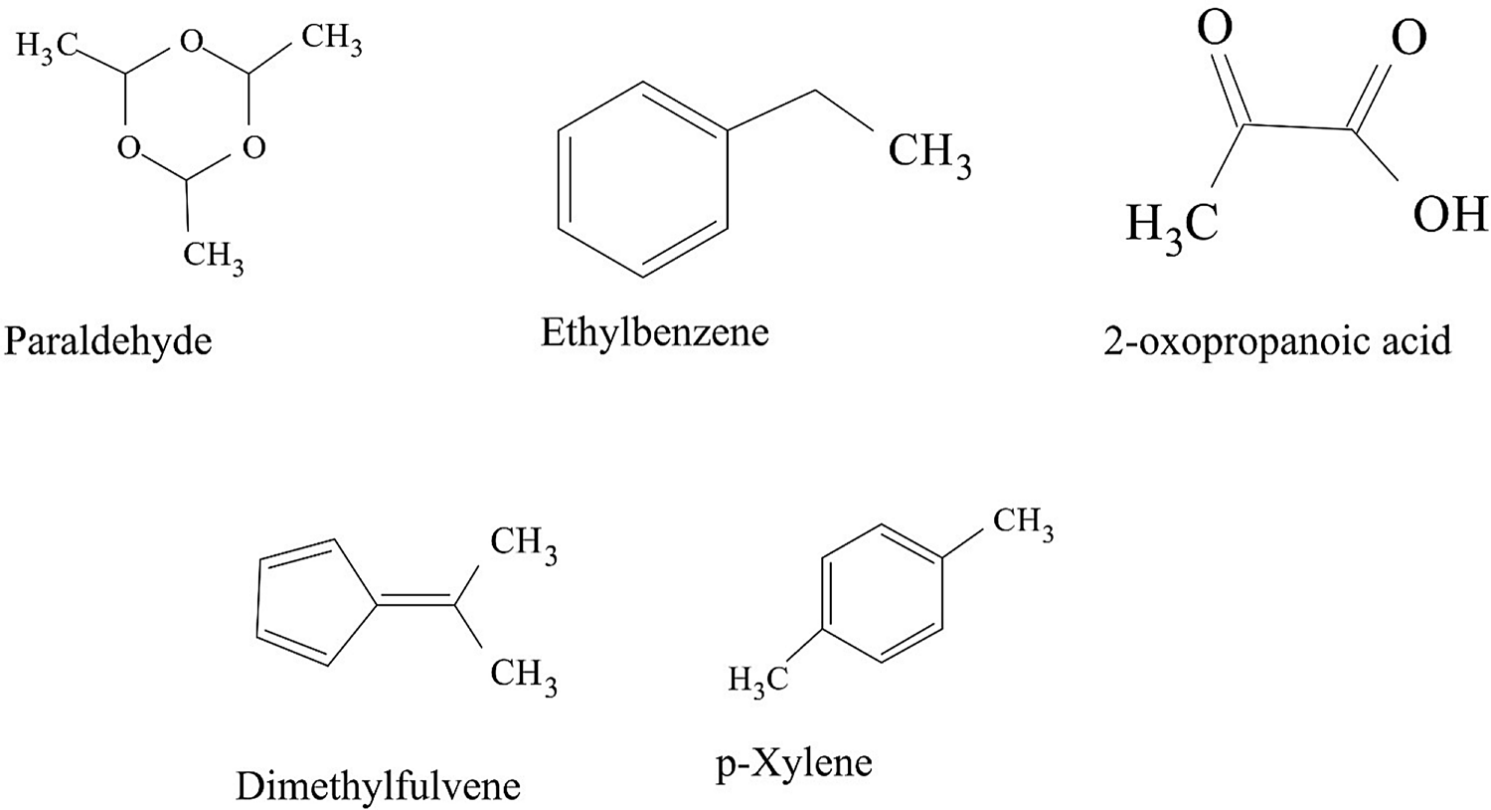
Structures of compounds from ethyl acetate fraction for all isolates

The characterized and identified VOCs from chloroform extract that is trichloromethane and that of ethyl acetate extract that is paraldehyde, ethylbenzene, dimethylfuvene and *p*-xylene had RT corresponding to 5.4, 3.46, 5.05, 5.35 and 6.14 respectively. Dimethylfulvene exhibited the highest intensity or peak area with 89.11% while trichloromethane exhibited the lowest with 27.37% and paraldehyde, ethylbenzene and *p*-xylene were 83.79%, 52.37% and 47.92% respectively (Table 2, Figs. 2, 3, 6, 7).

#### Hexane and methanol extract

**Fig. 8:**
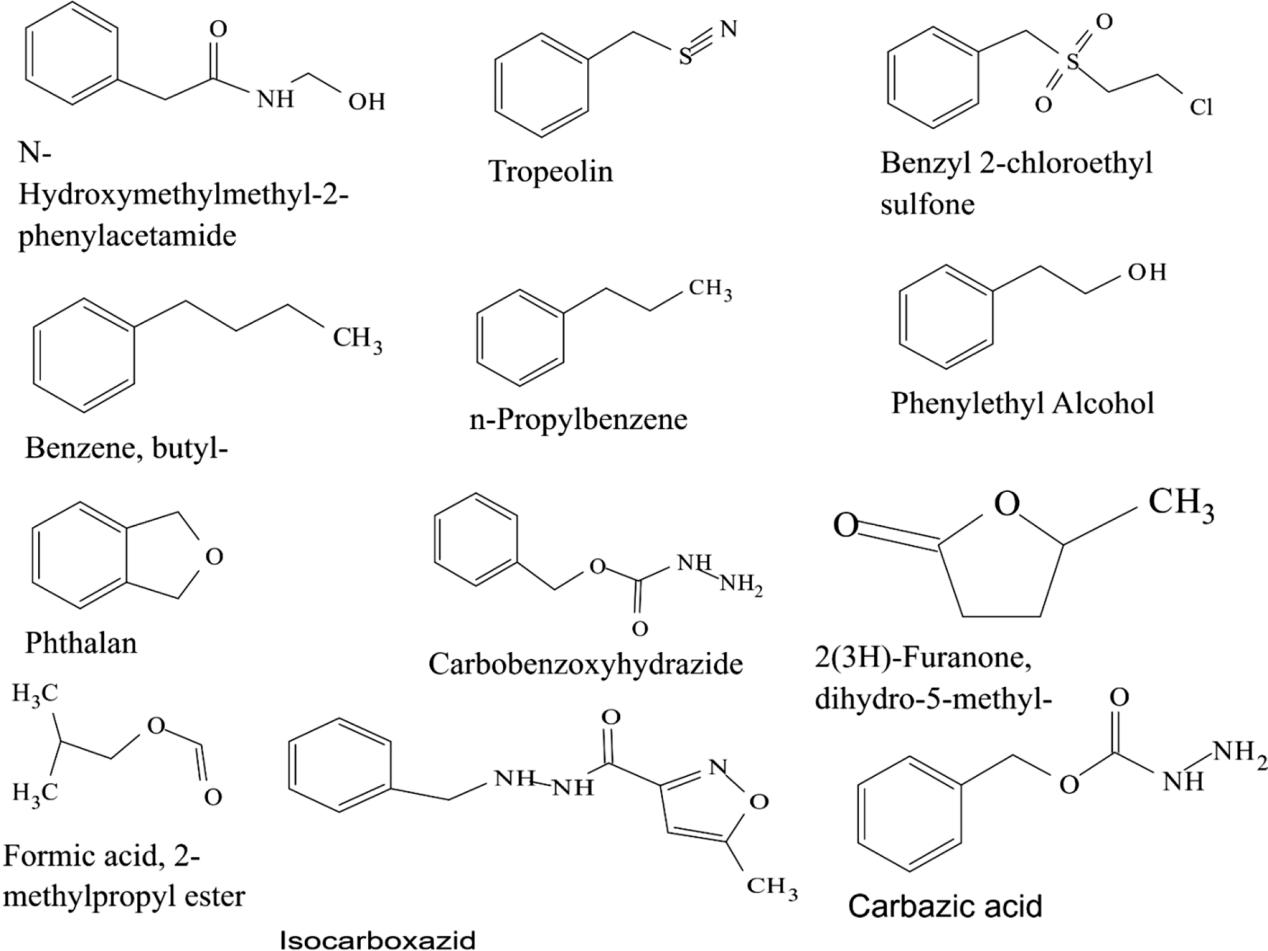
Structures of compounds from hexane fraction for all isolates

**Fig. 9:**
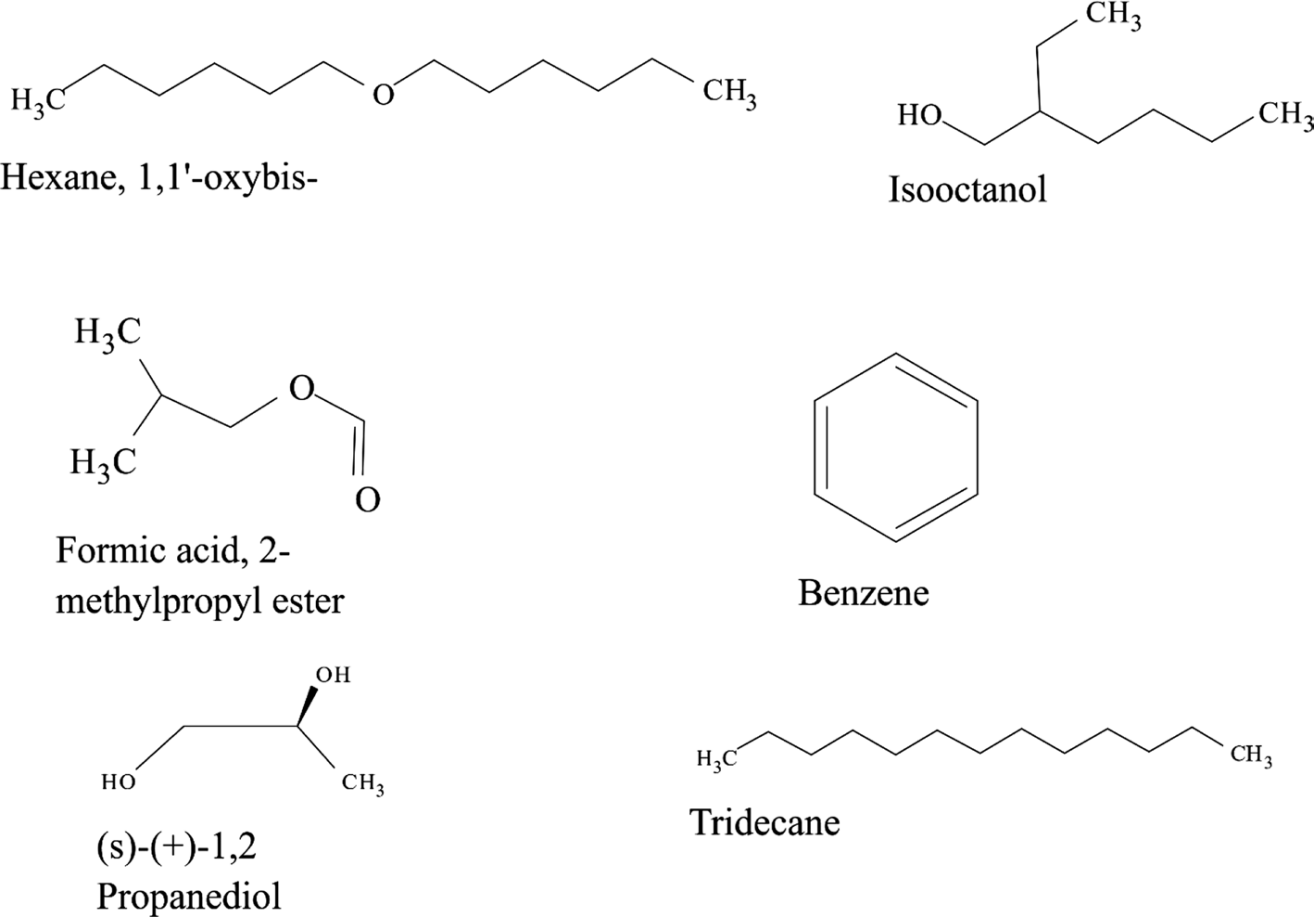
Structures of compounds from methanol fraction and petroleum ether for all isolates

The GC–MS chromatograms of hexane extract revealed that even though there were 6 peaks, only 4 peaks indicated the presence of secondary VOCs though the 2 peaks without VOCs presence were higher than some of the peaks with VOCs presence (100% and 39.94%). On comparison of the 4 peaks with the mass spectra from NIST library, the highest peak area of 86.72% and RT 3.06 picked 8 VOCs that were characterized and identified as phenylethylalcohol, benzyl 2-chloroethyl sulfone, *N*-hydroxylmethylmethyl-2-phenylacetamide, tropeolin, benzene, butyl-, *n*-propylbenezene, phthalan and carbobenzoxyhydrazide. The combination of VOCs at this specific RT and peak area was also noticed with the isolate *Bacillus* sp. using hexane extract and *B. thuringiensis* using methanol extract. The other 2 peaks were compared with the mass spectra from NIST library and were characterized and identified as 2(3H)-furanone dihydro-5-methyl and Formic acid 2-methylpropyl ester with the RT of 3.47 and 3.76 respectively and peak area of 9.65% and 6.25% respectively. The 4th peak also had a combination of VOCs ethylbenzene, carbazic acid, isocarboxazid with the RT of 5.05 and peak area of 32.72%. The GC–MS chromatogram of methanol extract showed 3 peaks out of which one of them though with peak area of 100% had no hit. The other 2 peaks indicated presence of VOCs that were compared with the NIST library and where characterized and identified as paraldehyde and s-(+)-1,2 propanediol. Retention time and peak area were highest in s-(+)-1,2 propanediol (3.47 and 71.53) while paraldehyde was 3.28 and 56.22% respectively (Table 2, Figs. 2, 3, 8, 9).

### Analysis of GC–MS chromatograms for *B. thuringiensis*

#### Benzene and butanol extract

**Fig. 10:**
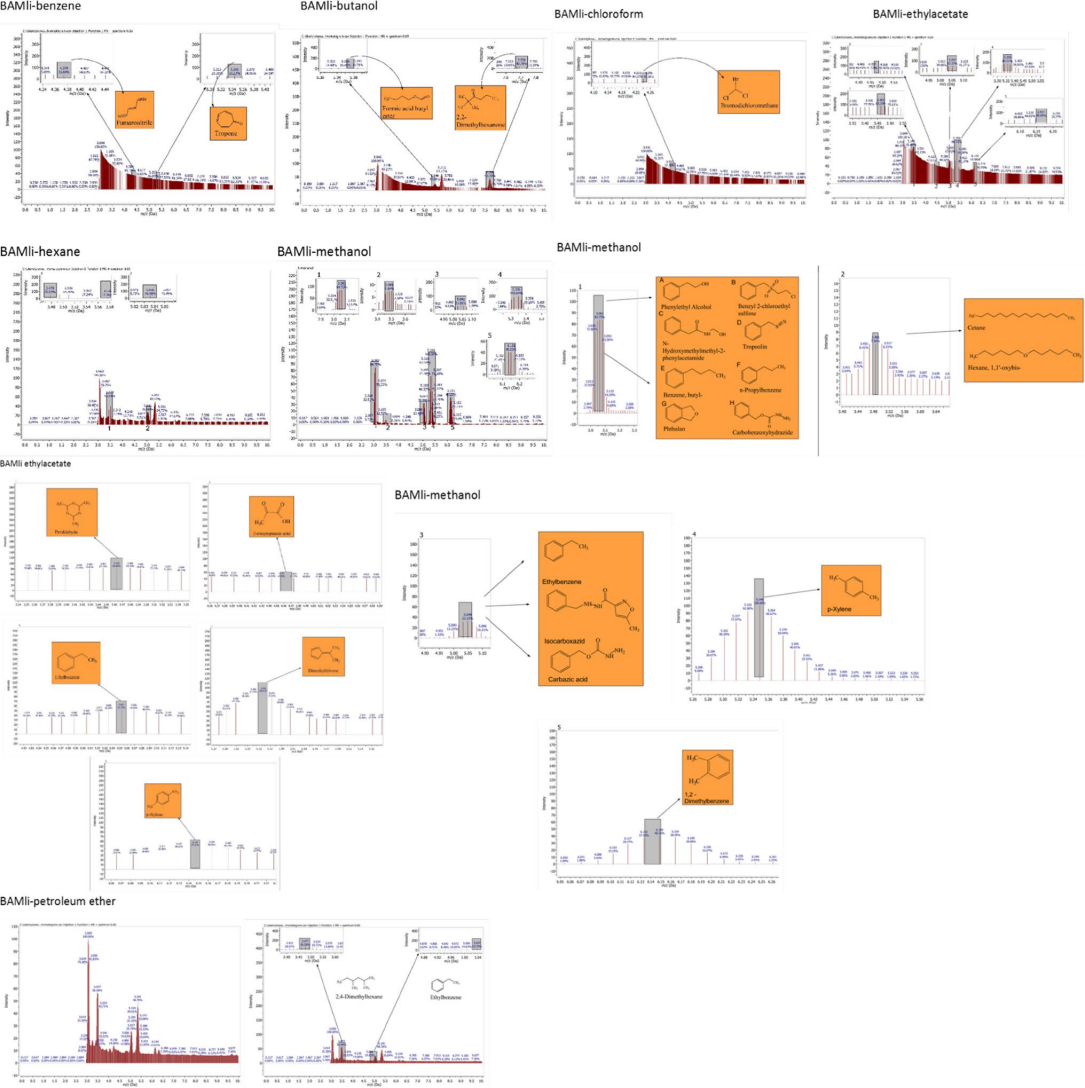
GC–MS chromatogram of *Bacillus thuringiensis* (BAMli) comparing extracts of benzene, butanol, chloroform, ethyl acetate, hexane, methanol and petroleum ether extract

The GC–MS chromatogram of benzene extract from *B. thuringiensis* did not show very sharp peaks but two of the peaks indicated the presence of VOCs. On comparison to the mass spectra from the NIST library, the compounds were characterized and identified as fumaronitrile and tropone (Figs. 4, 10) with RT of 4.37 and 5.34 respectively. While fumaronitrile had the highest peak area of 35.44% compared to tropone with 25.23%. Two peaks of GC–MS chromatograms of butanol extract from isolate *B. thuringiensis* were characterized and identified by comparing with the NIST library as formic acid butyl ester and 2, 2-dimethylhexanone (Fig. 5) in the RT of 5.36 and 7.70 respectively. The highest peak area of 43.70% was exhibited by 2, 2-dimethylhexanone while formic acid butyl ester showed the lowest peak area of 15.88% (Table 3, Figs. 4, 5, 10).

#### Chloroform and ethyl acetate extract

The GC–MS chromatogram of chloroform extract revealed only 1 peak which was characterized and identified as bromodichloromethane (Figs. 6, 10) from the NIST library. The RT and peak area were observed as 4.25, 43.38% respectively. The characterized and identified VOCs from ethyl acetate extract of *B. thuringiensis* which were observed as 5 peaks when compared with NIST library. The mass spectra corresponded with paraldehyde, 2-oxopropanoic acid, ethylbenzene, dimethylfulvene, *p*-xylene (Fig. 7) at RT 3.46, 4.45, 5.05, 5.35 and 6.14 respectively. The compound dimethylfulvene had the highest peak area of 89.11% while 2-oxopropanoic acid had the lowest at 41.55% (Table 3, Figs. 6, 7, 10).

#### Hexane and methanol extract

The characterized and identified VOCs from hexane extract of *B. thuringiensis* were observed as 3 major peaks (Figs. 8, 9, 10). When compared with NIST library, the mass spectra of the first peak corresponded to ethyl benzene (Fig. 10) in RT of 5.03 and peak area of 26.98%. The second peak corresponded to tridecylamine (Fig. 10) with 3.57 RT and peak area of 12.76%. The third peak was identified as combination of 2, 4-dimethylhexane and dihexylether (Fig. 10) in RT of 3.47 and peak area of 55.54%. GC–MS chromatogram of methanol extract showed 5 peaks with the first peak having a combination of VOCs as was mentioned with *B. amyloliquefaciens*, hexane extract (Figs. 8, 9, 10). The second peak also had a combination of 2 peaks identified as cetane and hexane, 1, 1′-oxybis and 3.85 in RT and peak area of 7.36%. The third peak was identified as a combination of ethylbenzene, isocarboxazid and carbazic acid with RT in 5.04 and peak area of 32.23%. The fourth peak corresponded to *p*-xylene in 5.34 RT and had the highest peak area of 100%. The fifth metabolite identified was 1, 2-dimethybenzene in 6.14 RT and 40.58% peak area (Table 3, Figs. 8, 9, 10).

### Petroleum ether extract

GC–MS chromatogram of petroleum ether extract showed 4 peaks with 2 peaks characterized and the metabolites identified as 2,4-Dimethylhexane and Ethylbenzene in RT 3.47 and 5.03 respectively. The highest peak area of 56.58% was exhibited by 2,4-Dimethylhexane while Ethylbenzene exhibited 25.79% peak area (Table 3, Figs. 9, 10).

### Analysis of GC–MS chromatograms for *Bacillus* sp.

#### Butanol and ethyl acetate extract

**Fig. 11:**
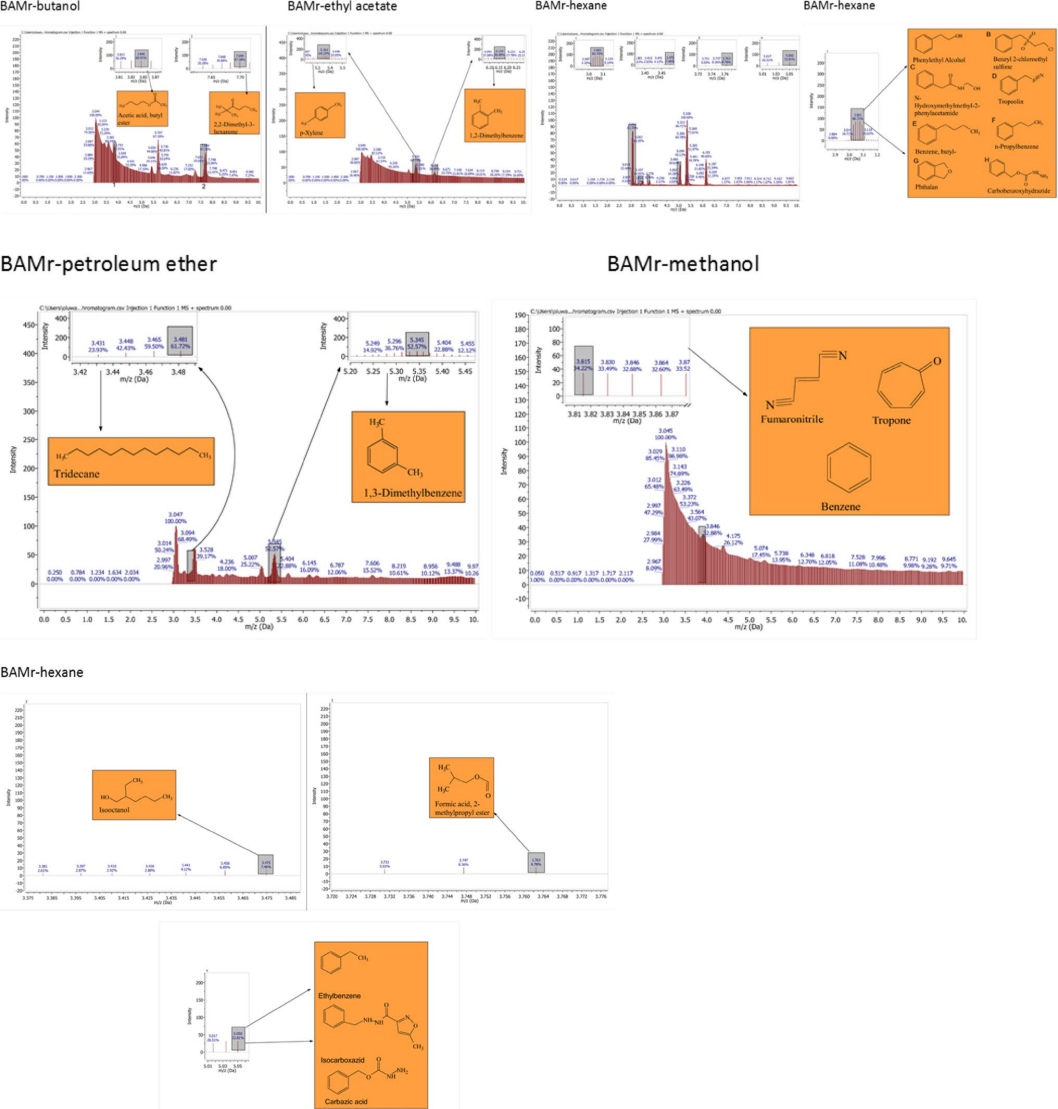
GC–MS chromatogram of *Bacillus* spp. (BAMr) comparing extracts using butanol, ethyl acetate, hexane, petroleum ether and methanol

The GC–MS chromatogram of butanol extract of isolate *Bacillus* sp. shows 2 sharp peaks, indicating the presence of VOCs characterized and identified as acetic acid, butyl ester and 2,2-dimethyl-3-hexanone (Table 4, Figs. 2, 5, 11) in RT 3.84 and 7.69 respectively. Acetic acid, butyl ester has the highest peak area of 60.41% while 2,2-dimethyl-3-hexanone has a peak area of 47.28%. The GC–MS chromatogram of ethyl acetate shows 2 peaks of characterized and identified VOCs *p*-xylene and 1,2-dimethylbenzene (Fig. 11) in RT of 5.35 and 6.15 respectively. The highest peak area of 64.33% was observed in *p*-xylene while1,2-dimethylbenzene had a peak area of 36.89% (Table 4, Figs. 2, 7, 11).

#### Hexane extract

Hexane extract produced GC–MS chromatograms of 4 peaks indicating the presence of microbial VOCs. The first peak had combination of VOCs as was mentioned in *B. amyloliquefaciens*, hexane extract and *B. thuringiensis* methanol extract in RT of 3.06 and the highest peak area of 86.72%. The third peak was identified as a combination of ethylbenzene, isocarboxazid and carbazic acid which was the same with the third peak of methanol extract of *B. thuringiensis* (Fig. 8) with RT in 5.05 and peak area of 32.23%. The remaining 2 peaks were characterized and identified as isooctanol and formic acid, 2-methylpropyl ester in RT 3.47 and 3.76 respectively. Isooctanol showed the lowest peak area of 7.46%, while formic acid, 2-methylpropyl ester also showed a low peak area of 8.78% (Table 4, Figs. 2, 8, 11).

#### Methanol and petroleum ether extract

The GC–MS chromatogram of petroleum ether extract showed 2 peaks indicating the presence of microbial VOCs. In comparison with the mass spectra of VOCs in the NIST library, the compounds were characterized and identified as tridecane and 1,3-dimethylbenzene (Figs. 9, Fig. 11) in RT of 3.48 and 5.34 respectively. Tridecane showed the highest peak area of 61.72% while 1,3-dimethylbenzene showed peak area value of 52.57%. Chromatogram from methanol extract only picked one peak indicating the presence of microbial VOCs which were characterized and identified as fumaronitrile, tropone and benzene (Figs. 9, 11) in RT 3.81 and peak area of 34.22% (Table 4, Figs. 2, 9, 11).

### Antibacterial potential of bioactive compounds/volatile organic compounds (VOCs)

**Fig. 12:**
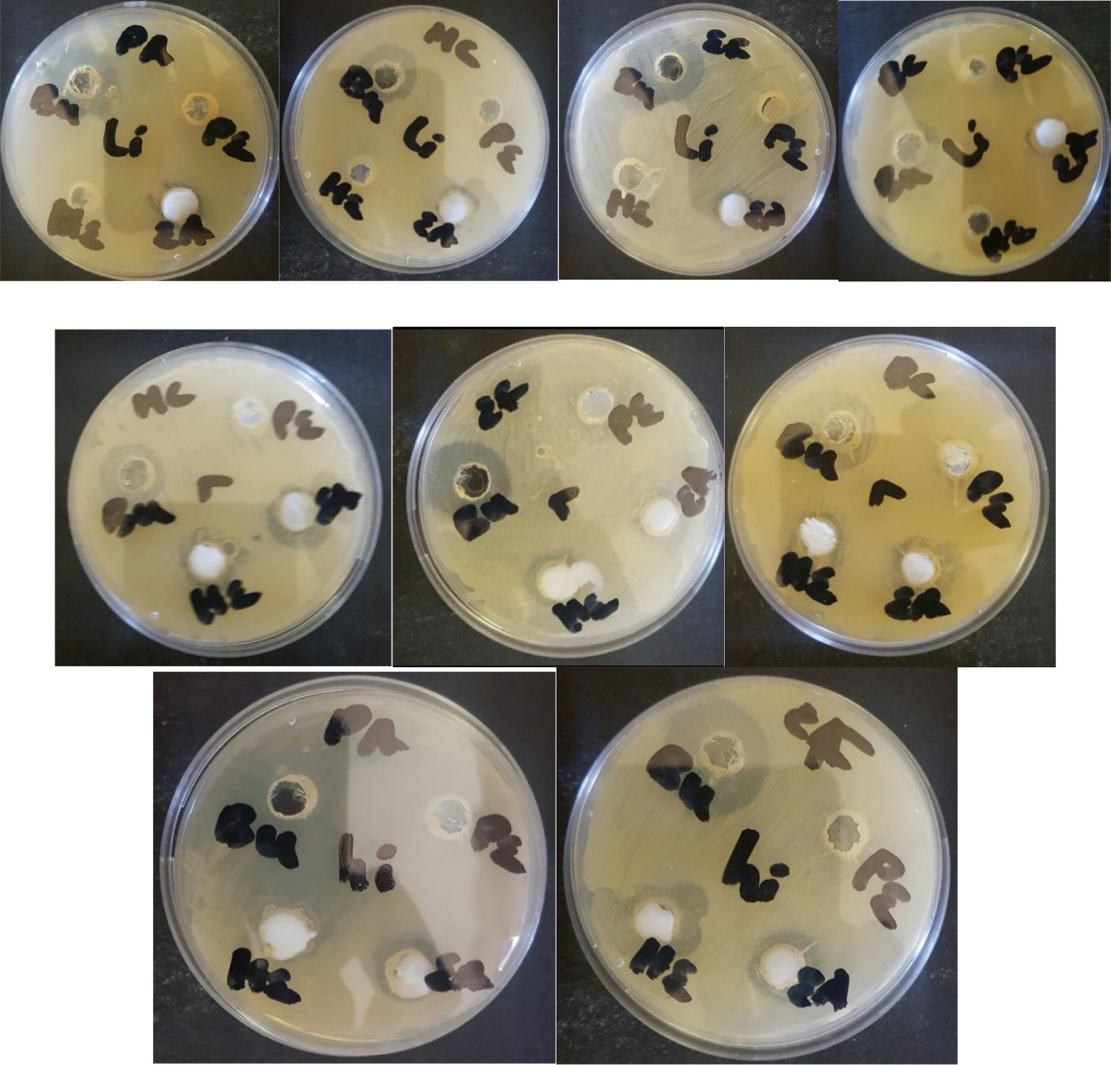
Antibacterial activities of crude extracts (*BU* Butanol, *PE* Petroleum ether, *EA* ethyl acetate, *HE* Hexane) of *Bacillus amyloliquefaciens* (hi), *Bacillus thuringiensis* (li) and *Bacillus* spp (r) against selected pathogens *B. cereus* (BC), *M. cryophilus* (MC), *P.aeruginosa* (PA) and *E. feacalis* (EF)

The organisms used in this assay are *B. cereus*, *P. aeruginosa*, *E. feacalis* and *M. cryophilus* which are locally isolated organisms (Sansinenea and Ortiz). *B. cereus* is a food poisoning pathogen worldwide with serious health implication, so it is very important in the food industry and agriculture (Tewari and Abdullah 2015). *E. feacalis* and *P. aeruginosa* are among the diverse type of bacteria capable of causing infection in man, animals and plants causing plants death 7 days post inoculation (Jha et al. 2005; Walker et al. 2004). *M. cryophilus* is a psychrophilic bacterium that is able to regulate lipid acyl chain length. (Kates 2012), this can also make it very resistant to biomolecules. The bacteria used for the tests were resistant to at least one known antimicrobial agent (Adegboye and Babalola 2013). Antagonistic assay against selected bacterial pathogens was carried out on the crude extracts of VOCs reveals that butanol extracts showed more antagonism against the selected pathogens (Fig. 12).

Butanol extract from *B. amyloliquefaciens* had 28 mm zone of inhibition against *B. cereus* compared to 18 mm and 16 mm by *Bacillus* sp. and *B. thuringiensis* respectively. It was also the highest with 24 mm zone of inhibition against *M. cryophilus* compared to 12 mm and 19 mm by *Bacillus* sp. and *B. thuringiensis* respectively. Butanol extract from *B. thuringiensis* was highly effective against *E. feacalis* and *P. aeruginosa* having 23 mm and 26 mm zones of inhibition respectively. This was also the highest compared to zones of inhibition from *Bacillus* sp. and *B. amyloliquefaciens* having 18 mm/15 mm and 21 mm/15 mm against *E. feacalis* and *P. aeruginosa* respectively. The hexane and ethyl acetate extract from *Bacillus* sp. were effective against *P. aeruginosa* with 12 mm and 17 mm zone of inhibition respectively compared to no zones of inhibition from hexane extract of *B. amyloliquefaciens* and *B. thuringiensis*. Zones of inhibition of 2 mm and 6 mm were observed against *P. aeruginosa* from ethyl acetate extract of *B. amyloliquefaciens* and *B. thuringiensis* respectively. Petroleum ether extract from bioactive compounds of isolate *B. amyloliquefaciens* had the least antagonism activity against test organisms, while the butanol extract of bioactive compounds from *B. amyloliquefaciens* had the highest antagonism activity against all test organisms (Table 5).

**Table 5:** Antibacterial activities of the active fractions against test organisms

Zone of inhibition (mm)												
Test organisms	Butanol			Hexane			Petroleum ether			Ethyl acetate		
	*Bacillus* sp.	*B. amyloliquefaciens*	*B. thuringiensis*	*Bacillus* sp.	*B. amyloliquefaciens*	*B. thuringiensis*	*Bacillus* sp.	*B. amyloliquefaciens*	*B. thuringiensis*	*Bacillus* sp.	*B. amyloliquefaciens*	*B. thuringiensis*
*E. Faecalis*	18	21	23	6	8	4	4	0	0	0	12	7
*B. Cereus*	18	28	16	6	12	8	0	0	6	12	8	4
*M. Cryophilus*	12	24	19	8	8	12	7	0	0	10	18	8
*P. Aeruginosa*	15	15	26	12	0	0	0	0	4	17	2	6

### The relevance of these data to the production of bioactive molecules by microbial cultures

Even though hexane had the highest number of bioactive metabolites produced, its antibacterial activities were not as inhibitory against test organisms as compared to that of butanol extract with only 6 different metabolites produced. It is also worthy of note that even though some of the solvents showed metabolites production in chloroform, methanol and petroleum ether, there were no antibacterial activities from their fractions based on zones of inhibition (Tables 2, 3, 4, Fig. 12). The relative abundance of the various bioactive compounds depicted by the peak area percentage revealed that dimethylfuvene from ethyl acetate extraction had the highest relative abundance with 89.11% while Formic acid 2-methylpropyl ester from hexane extraction had the lowest with 6.25%. Among the bioactive compounds produced by the three selected isolates were tridecane, acetic acid butyl ester, paraldehyde, s-(+)-1,2 propanediol, tropone, phthalan and *p*-xylene with relative abundance of 61.72, 60.41, 83.79, 71.53, 24.06, 86.72 and 64.33 respectively.

## Discussion

Rhizobacteria have been observed to produce secondary metabolites especially antibiotics (Raaijmakers and Mazzola 2012). Gas Chromatography-Mass Spectrometry (GC–MS) analysis of crude extract from the 3 isolates in this study detected 68 compounds using 7 extraction solvents while *Streptomyces* spp. isolated from the rhizosphere of chili pepper was observed to detect 77 compounds using ethyl acetate and based on RT and peak area percentage (Jalaluldeen et al. 2015). A total of 22 volatile compounds were secreted by 6 bacterial strains from the rhizosphere of lemon plant (Gutiérrez-Luna et al. 2010) while a total of 38 volatiles were released by 4 PGPR strains and were used to trigger growth in *Arabidopsis thaliana* (Farag et al. 2006). This difference in bioactive compounds detected could be as a result of the volatility of the compounds or extraction solvent (Groenhagen et al. 2013). Rhizobacterial strains very close to *B. thuringiensis*, *B. amyloliquefaciens* and *Bacillus* sp. have been involved in the production of one or more bioactive compounds (Arguelles-Arias et al. 2009; Bacon et al. 2015; Baysal et al. 2008). *Bacillus* spp. are able to withstand harsh environmental conditions in the soil because of the production of spores that are able to withstand extreme weather conditions. They are also able to compete favourably with other microbes as a result of VOCs production (Amin et al. 2015). *Bacillus* spp. have been observed to be able to produce diverse VOCs which are effective against human beings, plant and animal pathogens (Kai et al. 2007).

Antagonistic activities have been reported from many origins including bacteriocins, enzymes and volatiles while the effect of volatiles have been understudied but not given the relevant attention it deserves. In this study, it was shown that the rhizosphere of Bambara groundnut was able to attract organisms which could be important pesticide-producing agents as well as useful in drug production and other industries. The GC–MS result from the three isolates showed that mostly the same compound was present and this could be attributed to the three organisms being members of the same genus.

According to Gao et al. (2018), benzothiazole and 4-di-tert-butylthiophenol were key inhibitory VOCs produced by *B. subtilis* CF-3. They were able to significantly inhibit the growth of postharvent fungi *Monilinia fructicola* and *Colletotrichum gloeosporioide* with peak of 73.46% and 63.63% respectively. This peak is close to s-( +)-1,2 propanediol produced by *B. amyloliqefcaiens* with peak 71.53.

Eight *Bacillus* strains showed the presence of ketones which included 3-methyl-2-pentanone, 2-heptanone, 2-octanone, 2-decanone, 5-methyl-2-hexanone, 2-nonanone, 2-dodecanone, 2-undecanone and 5-methyl-2-heptanoneand2-pentanone. They are rich resources of bioactive volatiles and were found to be effective in inhibiting growth of *Fusarium solani* (Li et al. 2015).

Four *Bacillus amyloliquefaciens* subsp. *Plantarum* strains UCMB5033, UCMB5036, UCMB5113 and FZB42 were inoculated with fungal strains *Botrytis cinerea* 30,158, *Alternaria brassicicola* 20,297, *A. brassicae* 980:3, *Sclerotinia sclerotiorum* 13 MM and *Verticillum longisporum* D11 which are phytopathogens. Some of the VOCs produced were 2,3butanedione, acetoin, 5-methyl-heptanone, 2-methylpyridine, 2-pentanone, 2-heptanone and 3-methylbutanol. The study revealed that *Bacillus* VOCs anatagonised several fungal growth at varied degrees (Asari et al. 2016).

 It  was observed that *Fusarium oxysporum* f. sp. *Cucumerinum* J. H. Owen (FOC)-infected cucumber seedlings secreted more citric acid and fumaric acid, which stimulated the chemotaxis response and biofilm formation of *B. Amyloliquefaciens* SQR9, and thus enhanced the root colonization of *B. Amyloliquefaciens* SQR9 and suppressed disease infection (Liu et al. 2014).

*Bacillus subtilis* FA26 was evaluated for its antibacterial activities against *Clavibacter michiganesis* ssp. *Sepedonicus* (Cms), the causal agent of bacterial ring rot of potato. It was observed that *B. subtilis* FA26 produced 11 VOCs with 4 of them significantly active against Cms (benzaldehyde, nonanal, benzothiazole and acetophenone) (Rajer et al. 2017).

From the 36 volatile compounds detected from *Bacillus amyloliquefaciens* NJN 6, 11 produced volatile compounds (VOCs) that inhibit the growth and spore germination of *Fusarium oxysporum* f.sp. Cubense completely. They included 2-undecanone,2-dodecanone,and2-tridecanone, benzothiazoles phenol and 2,3,6-trimethylphenol (Yuan et al. 2012).

*Bacillus atrophaeus* CAB-1 was able to suppress cucumber powdery mildew and tomato gray mold activity using lipopeptides, a novel protein and volatile compounds. The volatile compounds produced by CAB-1 were complex, including a range of alcohols, phenols, amines, and alkane amides. O-anisaldehyde represented one of the most abundant volatiles with the highest inhibition on the mycelial growth of *B. cinerea* (Zhang et al. 2013).

Contained in a number of pesticides and antidepressants are notable compounds produced such as 1,3-dihydroisobenzofurans, commonly known as Phthalan (extracted also in this study). It is comprised of a large number of bioactive compounds in which some of them are antihistaminic, antifungal, antisecretory and antidepressive (Karmarkar et al. 2014). This compound has been synthesized from plants and other compounds and have been commercialised also but so far not from rhizobacteria as documented in this study.

It was observed that the bioactive compounds extracted using butanol were the potent antibacterial compounds from the isolates. They showed considerably larger zone of inhibition compared to other selected solvent extracts while the least zones were recorded in the petroleum extract solvents (Table 3). One solvent performs better than the other because of the difference in polarity. Butanol extract may show antagonism against some selected pathogens because it has four carbon atoms in its structure and allows to penetrate into the cell walls compared with ethanol that has two carbon atoms and the other solvents. Antimicrobial activities increase as the polarity of the solvents increase (Parekh and Chanda 2007). Also, according Bakht et al. (2011), butanol extract also had the highest activity against bacterial pathogen *Erwinia carotovora*.

The GC–MS result is in the order hexane > methanol > ethyl acetate > butanol > benzene and petroleum ether while chloroform < petroleum ether (Figs. 2, 3, 4, 5, 6). Benzene is less than petroleum ether while chloroform had the least number of notable compounds.

Some of the bioactive compounds like tropolone have been shown to have a strong inhibitory activity on plant growth and are known to have antimicrobial and insecticidal activity. Troplone are found in *Cupressaceae* trees, such as *Chamaecyparis obtusa*, *Thuja plicata*, *Thujopsis dolabrata* var. *Hondai*, *Hiba arboruitae*, *Cupressus lusitanica*. It reduced the production of ethylene in peach seeds that were excised and also inhibited ethylene production by suppressing the production of 1-aminocyclopropane-1-carboxylate (ACC) synthase and oxidase in the mesocarp of wounded winter squash. Strong antifungal activity and broad antimicrobial spectrum of tropolone have led to their wide utilization in agriculture, clinical products, cosmetics and other areas (Saniewski et al. 2007). They are able to chelate iron and make it available for plant growth promotion (Saniewski et al. 2007). Another compound, *p*-xylene which is a chemical feedstock in the chemical industry was secreted by the three selected isolates, these were all *Bacillus* spp. Other bioactive compounds were tridecane, fumaronitrile, isocarboxazid and tridecylamine.

The data from the different solvents used was able to clarify the fact that more bioactive compound can be synthesized from rhizobacteria if the appropriate solvents are used. In this study, Hexane and Butanol where able to extract more bioactive compounds compared to the other solvents used. But Butanol extracts had the highest antagonistic activity against test organisms. The butanol extract from the 3 bacillus isolates were more effective antimicrobials compared with the other extracts. The compounds in the extracts includes 2, 2-dimethylhexanone, acetic acid,butylester, 2,2-dimethyl-3-hexanone, Formic acid butyl ester and 2,2-dimethylhexane-3-one. The study by Hossain et al. (2013), revealed that 2, 2,-dimethylhexane-3-one was part of the bioactive compounds from the chloroform crude extract of the medicinal plant *Datura metel*, though its antibacterial properties was not within the scope of the study. This data opens door to more research on application of the use of hexane, ethyl acetate and butanol as a more effect solvent for extraction of more bioactive compounds and also more antagonistic activities respectively. The data presented in this study reveals that there are untapped bioactive compounds in soil microorganisms in the rhizosphere of legumes and in particular Bambara groundnut.

## Conclusion

Apart from antibiotics, VOCs are another class of secondary metabolites that have showed great promise in combating pathogenic organisms. The VOCs crude extracts from this study were antagonistic against test organisms, thus, making them good antibacterial compounds for the agricultural, medical and pharmaceutical industries. These three isolates (*Bacillus* sp., *B. thuringiensis* and *B. amyloliquefaciens*) are good sources of bioactive compounds and the compounds can be modelled into antibacterial agents. Since not much work has been done to exploit these compounds, it means basic research should be conducted more to exploit the hidden potentials of these compounds and the organisms producing them as this might lead to the discovery of new world of metabolism in bacteria. This is going to be the next stage of this research in which case data from this study will be used to determine which compounds are most likely to be responsible for antimicrobial activity in the data presented here, and those most promising antimicrobial compounds can be the subject of future work to prove their antimicrobial activity and to optimize production.
